# Effects of Habitat Types on Macroinvertebrates Assemblages Structure: Case Study of Sun Island Bund Wetland

**DOI:** 10.1155/2019/2650678

**Published:** 2019-02-13

**Authors:** Nagam Khudhair, Cai Yan, Manhong Liu, Hongxian Yu

**Affiliations:** ^1^College of Wildlife Resources, Northeast Forestry University, Harbin 150040, China; ^2^Biology Department, Education College for Women, University Of Anbar, Ramadi 31001, Iraq

## Abstract

Sun Island Bund Wetland (SIBW) is a river floodplain wetland located at the south part of Heilongjiang Province in Northeast China. An investigation of the influence of habitat type on macroinvertebrates assemblages structure was conducted in July 2016. Nine (9) sampling sites were selected based on sediment type, water condition, and aquatic vegetation type. Macroinvertebrates attributes including density, biomass, and four diversity indices (Simpson diversity index, Margalef richness index, Shannon-Weiner index, and Pielou evenness index) were assessed. A total of 53 taxa were collected during the study period, with the highest density dominated being from aquatic insects and gastropods.* Bellamya purificata* and* Exopalaemon annandalei* were the most dominant among all the species. The results showed that the assemblages structure of macroinvertebrates in different habitats was significantly different. Also, the results with PCA showed that the higher values of invertebrates density, biomass, diversity indices, and species richness had a greater association with the habitat types of silt-humus sediment, closed lentic area, and submerged-flouting-emergent vegetation.

## 1. Introduction

Habitat structure is a key factor determining the occurrence and distribution of macroinvertebrates in freshwater ecosystems [1]. In rivers, where the physical structure is a major feature, recognition of the potential importance of habitat structure to benthic organisms is long-standing [2]. River macroinvertebrates have been shown to be influenced by both habitat complexity and heterogeneity [3], and structural features have consequently become a central focus in river management and restoration [4, 5]. Several studies have attempted to relate environmental factors such as sediment type, vegetation type, and physical and chemical parameters to influence macroinvertebrates in aquatic ecosystems mainly lakes and rivers globally [6–9]. Several macroinvertebrate species develop various morphological and physiological adaptations strongly associated with habitat conditions such as the type, composition, and size of the substrate composition in streams [10]. The substrate size can vary from larger and more complex, such as pebbles, leaves, and woody materials that support a great diversity and abundance, to fine sediments like sand, with few species [11]. The substrate provides places for food and refuge for macroinvertebrates [11]. In addition to their ecological role, macroinvertebrates have been used by scientists as bioindicators of environmental quality in aquatic ecosystems because of their ubiquity, limited mobility, large size, abundance, and measurable duration of life cycles [12].

The contribution of aquatic macrophytes to the structure and function of freshwater habitats has long been recognized [13]. In wetlands, a well-developed macrophyte community provides shelter against vertebrate predation of vulnerable prey species such as macroinvertebrates and small fish [14]. In addition, macrophytes provide more surface area attachment for periphyton, a major component in the diet of macroinvertebrate primary consumers [14, 15]. Therefore, macrophytes influence the diversity, abundance, and distribution pattern of aquatic invertebrates and vertebrates. Hicks [16] noted differences in the composition of macroinvertebrate assemblages composition within water soldier (*Stratiotes aloides*) beds in relation to successional gradient of submerged versus floating plants. Different aquatic systems can have different environmental gradients and habitats which may regulate macroinvertebrate assemblage structure. In the present study, we investigated macroinvertebrate community structure in Sun Island Bund Wetland (SIBW) which is a river floodplain wetland located in the south part of Heilongjiang Province of Northeast China. Recently, attention has been given to the aquatic vegetation (*Phragmites australis*) and microorganisms (*Arbuscular Mycorrhizal* Fungi) [17, 18]. However, how the structure of macroinvertebrate assemblages may be influenced by habitat types has not been studied. Therefore, the objective of this study was to analyze the structure of macroinvertebrate assemblages among different habitat types of SIBW.

## 2. Materials and Method

### 2.1. Study Area

**Figure 1:**
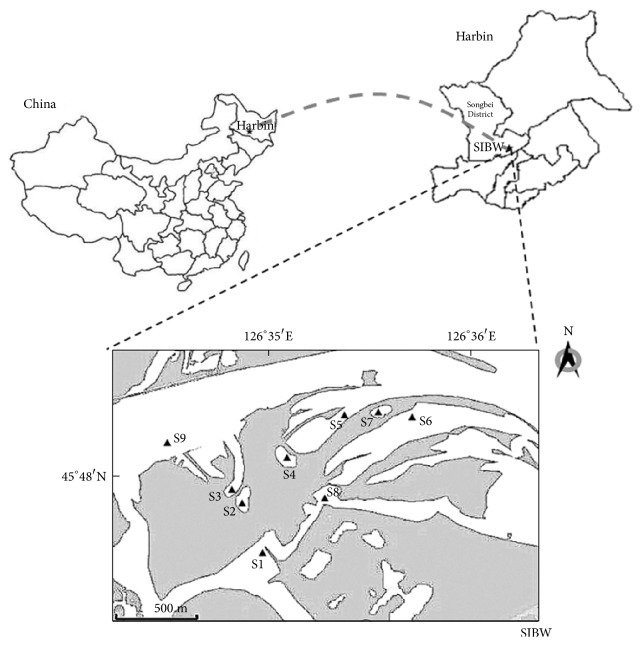
Map of the study area. Top left is the map of China; right is the map of Harbin and below is the study area SIBW. Letter and number (S1, S2, S3, S4, S5, S6, S7, S8, and S9) represent the sampling sites. More details of the sampling sites are provided in Table 1.

The study was conducted in a Sun Island Bund Wetland (SIBW) which is a river floodplain wetland (45°41′N- 45°47′N and 126°31′E -126°36′E) located in the south part of Heilongjiang Province of Northeast China (Figure 1). The study area is in the temperate continental monsoon climate zone with a mean annual temperature of 5.3°C. January and July are the coldest and hottest month with the annual mean temperature of -19.2°C and 22.8°C, respectively. The annual average precipitation is 523.30 mm [19]. SIBW comprises floodplains, marsh, hillock, water pools, flat terrain, and gently sloping among many others. The main types of vegetation are hydrophytes and phreatophyte [18].

SIBW is one of the important regions of wild animals and plants. It can provide a variety of ecological service functions of social economic value, such as the provision of fresh water resources, replenishment of groundwater, maintenance of regional water balance, regulation of local microclimate, control of soil erosion, mitigation of droughts and floods, degradation of pollutants, purification of the environment.

### 2.2. Field Sampling and Data Processing

The sampling was conducted in summer July 2016. The selection of the sampling sites was done to capture the effects of different habitat types on macroinvertebrates assemblages structure in the SIBW. Sampling sites were classified based on sediment type, water state habitat type, and composition of aquatic vascular vegetation (Table 1).

Prior to the collection of macroinvertebrates samples, water temperature (WT), pH, and oxidation-reduction potential (Eh) were measured and recorded in situ (HANNA, Hi8424). Water samples were also collected for the laboratory analysis of total phosphorus (PO_4_^3−^), total nitrogen (N), ammonia nitrogen (NH_4_+), and nitrite (NO_2_-) (HANNA, DRB200 & DR1900). Macroinvertebrates specimens were sampled using a D-net frame (30 cm aperture, 425*μ*m mesh), by sweeping through the water column in the shallow water until totaling an area of approximately 1 m^2^ was sampled. Collecting methods using a D-net frame was according to Maul et al. [20]. Aquatic macroinvertebrates specimens were collected from a variety of substrates at each sampling site including sand and gravel bed materials, stone and rocks (riprap), leaf packs, and coarse particulate organic matter with a D-net frame. A qualitative multihabitat composite sample was also taken. All specimens were separated from the sand, mud, and substrates by hand. Peterson grab (effective area: 0.0625 m^2^) was used for sampling in the sediments and deep water, and this method adapts to silt or humus sediment.

In addition to the D-net frame and Peterson grab sampling method, artificial trapping bags were created for sampling macroinvertebrates. The trapping bags were rectangular in shape 40*∗*20 cm (1 mm mesh size) filled with leaves and stones placed in the water for the organisms to colonize. After 14 days, the bags were retrieved. The samples were washed through a 425*μ*m mesh sieve to separate the organisms from extraneous materials and preserved in 85% alcohol and then transported to the laboratory for further analysis. In the laboratory, the macroinvertebrate communities were keyed to species or genus and counted using identification keys of Morse et al. [21]; Merritt et al. [22]; Dudgeon [23]; Thorp and Covich [24]; and Duan et al. [25].

Five biodiversity indices, including Species dominance index (y), Simpson diversity index (D), Margalef richness index (d), Shannon-Wiener index (H′), and Pielou evenness index (J), were used to describe the assemblages structure of macroinvertebrates. They were calculated as follows:

(1) y=fi×Pi,D=1∑Pi2,d=S−1ln⁡N,H′=−∑Piln⁡Pi,J=H′ln⁡S,

fi: the frequency of occurrence of species (i); Pi: proportional abundance of a given species (i); S: the total number of species; N: the total number of individuals of all species; y>0.02 represents species (i) which is the dominant species. There are statistics of biomass and density of macroinvertebrates using SPASS 16.0.

Physicochemical parameters were expressed as means and standard deviation (SD) for each sampling site. One-way analysis of variance (ANOVA) and Principal Component Analysis (PCA) with XLSTAT were used to test differences in species density, diversity, and richness among sampling sites. Before applying the parametric tests, the data were tested for homogeneity of variances using F-max test. This test was meant to decide if the difference between two or more sample variables is so small that it may be ignored. Least Significant Difference test (LSD, *α* = 0.05) was applied for multiple comparisons of means whenever analysis of variance resulted in significant F-values.

## 3. Results

### 3.1. The Physic-Chemical Parameters along Different Habitat Types in SIBW

From the results, the physic-chemical variables measured in situ did not vary based on sediment types. However, total nitrogen (N) and ammonia nitrogen (NH_4_^+^) differ among the sites based on vegetation type and water state (Table 2).

### 3.2. The Structure and Composition of Macroinvertebrates along Different Habitat Types

The species list of macroinvertebrates obtained in samples from all sampling sites in SIBW is presented in (Table 3); the macroinvertebrates collected were from 4 classes representing 12 orders, 21 families, and 53 taxa. The macroinvertebrates were divided into the aquatic insect, mollusk, annelid, and crustacean. Aquatic insects were most caught with 12 families, such as Chironomidae, Belostomatidae, Nepidae, Gomphidae, Sericostomatidae, Polycentropodidae, Hydropsychidae, Rhyacophilidae, Ephemeridae, Heptageniidae, Ephemerellidae, and Leptophlebiidae. In general, Chironomids were the most diverse and abundant family, which possessed 18 taxa followed by Gomphidae with 2 taxa. The aquatic insects had the highest number of species, contributing more than 56% of the total taxa (30 out of 53 taxa), followed by mollusks with 30.1% (16 out of 53 taxa). Annelid and crustacean accounted for 7.5 and 5.6% of the total species, respectively. Using the species dominance index,* Bellamya purificata* (Gastropoda) and* Exopalaemon annandalei* (Crustacea) were found to be the dominant species with values of 0.03 and 0.02, respectively. Species which were widely distributed in the SIBW are* Exopalaemon annandalei *(Crustacea),* Radix swinhoei*,* Bellamya purificata* (Gastropoda), and* Cricotopus albiforceps* (Chironomidae).

### 3.3. Macroinvertebrates Abundance and Diversity

Our results showed that the average density of the macroinvertebrates in the SIBW was 340.88 ind. /m^2^, while the average biomasses were 390.24 g/m^2^. The mean values of the Simpson diversity index (D), Margalef richness index (d), Shannon-Weiner index (H′), and Pielou evenness index (J) were 6.156, 1.284, 1.783, and 0.934, respectively (Table 4).

### 3.4. Effects of Sediment on Abundance of Macroinvertebrates in SIBW

**Figure 2:**
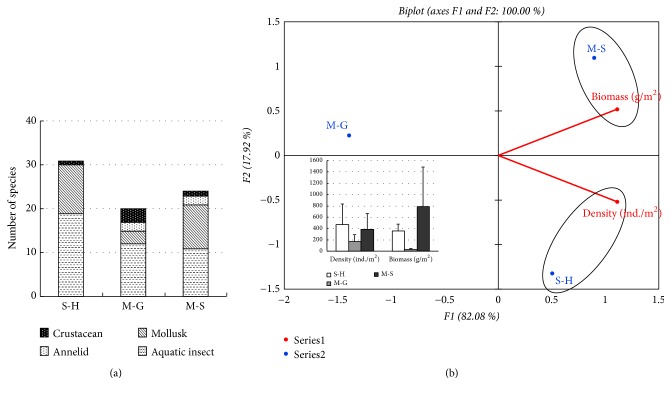
Effects of three types of sediment on macroinvertebrates distribution in the SIBW. (a) The numbering of macroinvertebrates shows the mollusks were greater in (S-H). (b) Principal component analysis (PCA) based on the density and biomass of total macroinvertebrates in different sediment. The density of macroinvertebrates had a greater association with the S-H sediment and the M-S sediment had the least association. The largest biomass of macroinvertebrates in M-S, sediment. S-H, Silt-humus; M-G, Mud-gravel; M-S, Mud-sand.

In general, the aquatic insects had the highest numbers of taxa followed by mollusks in the different types of sediment.  Figure 2(a) showed the mollusks were found in each sediment type. Silt-humus sediment (S-H) had 31 taxa, mud-sand sediment (M-S) had 24 taxa, and mud-gravel sediment (M-G) had 20 taxa. The results PCA showed that the density of macroinvertebrates had a greater association with the silt-humus sediment (472.3±364.5 ind./m^2^), followed by the mud-sand sediment (385.6±280.7 ind./m^2^), and the mud-gravel sediment (164.6±126.2 ind./m^2^) had the least association. Moreover, mud-sand sediment had the largest biomass of macroinvertebrates (781.5±706.8g/m^2^), followed by the silt-humus sediment (358.0±114.4g/m^2^), and then mud-gravel sediment (31.0±15.1g/m^2^) (Figure 2(b)). The highest biomass recorded in a mud-sand sediment was attributed to the high number of mollusks. Only Pielou indices (J) differ among the sites (Table 5).

**Figure 3:**
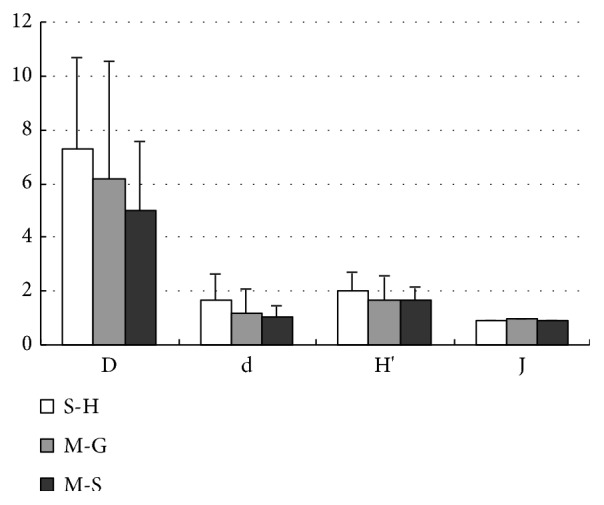
Biological index of different sediment habitats. D, Simpson diversity index; d, Margalef richness index; H′, Shannon-Weiner diversity index; J, Pielou evenness index. S-H, Silt-humus; M-G, Mud-gravel; M-S, Mud-sand.

The results of Figure 3 showed the biological index for different sediment habitats. It was quite clear that for the Simpson diversity index (D), S-H recorded the highest value followed by M-G and M-S. The trend was similar to a Margalef richness index (d) and Shannon-Weiner diversity index (H′). However, for Pielou evenness index (J), mud-gravel sediment had a slightly higher value, followed by mud-sand sediment and then silt-humus sediment.

### 3.5. Effects of Water State on Macroinvertebrates

**Figure 4:**
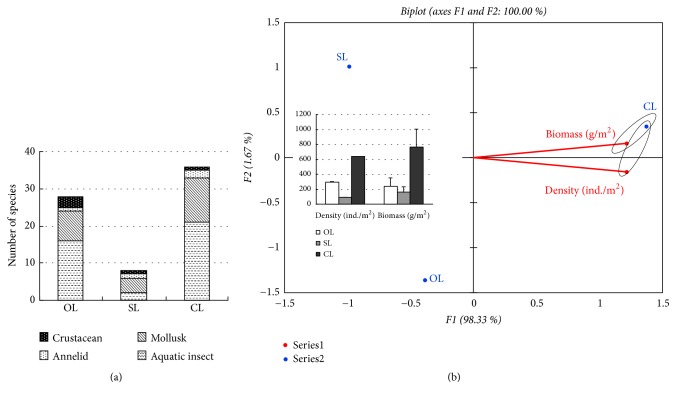
The effects of three types of water state on distribution macroinvertebrates in the SIBW. (a) The numbering of macroinvertebrates (mean ± SD). In the closed lentic area and the open lotic area, the aquatic insects were dominant followed by mollusks. While the seasonal lotic area was dominated by mollusks > aquatic insects > annelids and crustaceans. (b) Principal component analysis (PCA) of the density and biomass of total macroinvertebrates in different sediment, where the density and biomass of macroinvertebrates are almost a similar trend with CL having the highest values followed by OL and then SL. S-H, Silt-humus; M-G, Mud-gravel; M-S, Mud-sand.

Based on the water state type, closed lentic area (CL) recorded the highest number of species (36 taxa) (Figure 4). Open lotic area (OL) had 28 species less than closed lentic area while the seasonal lotic area (SL) registered only 8 taxa of macroinvertebrates. The aquatic insects were dominant in the closed lentic area and the open lotic area, followed by mollusks. The seasonal lotic area was dominated by mollusks followed by aquatic insects, annelids, and crustaceans (Figure 4(a)). The PCA showed the best conditions in relation to the water state, where the density and biomass of macroinvertebrates in the water state habitat types displayed almost a similar trend with CL having the highest values followed by OL and then SL (Figure 4(b)).

**Figure 5:**
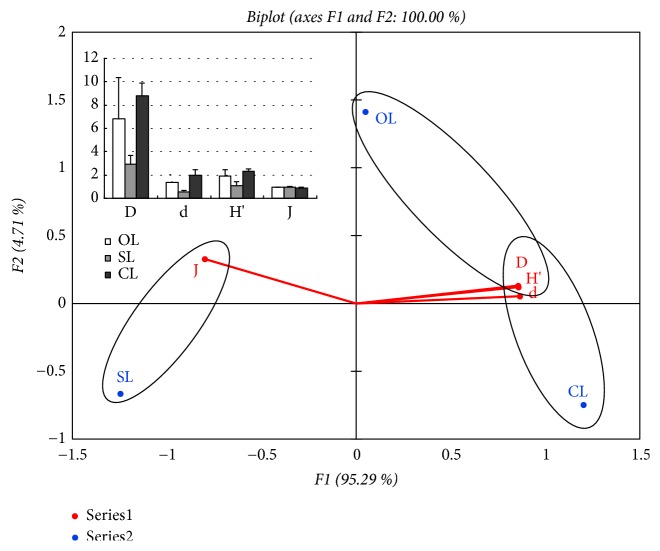
Principal component analysis (PCA) of biological indices comparison between three types of water. CL had the highest values of the Simpson diversity index (D), Margalef richness index (d), and Shannon-Weiner diversity index (H′). This was followed by OL and SL in the same order of indices. OL, Open lotic; SL, Seasonal lotic; CL, Closed lentic. D, Simpson diversity index; d, Margalef richness index; H′, Shannon-Weiner diversity index; J, Pielou evenness index. S-H, Silt-humus; M-G, Mud-gravel; M-S, Mud-sand.

Figure 5 and Table 6 present the results of biological indices assessed in the different habitats based on the status of water. With exception macroinvertebrate biomass and the Pielou evenness index (J), all macroinvertebrates attributes assessed differed among the sites at 95% confidence level. Notably, CL had the highest values of the Simpson diversity index (D), Margalef richness index (d), and Shannon-Weiner diversity index (H′). This was followed by OL and SL in the same order. There was no clear difference in the Pielou evenness index values for all three sites.

### 3.6. Effects of Vegetation Composition on Macroinvertebrates

**Figure 6:**
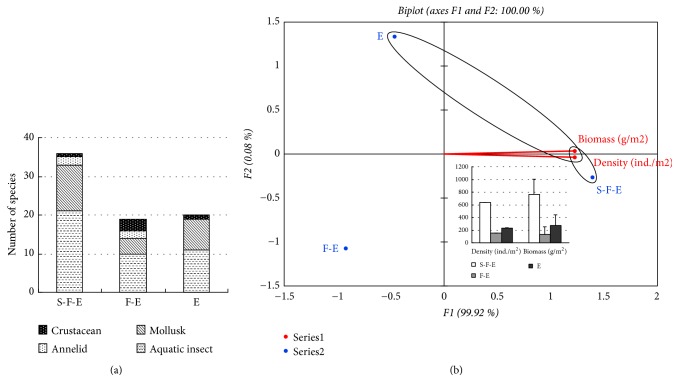
The effects of three types of vegetation to distribution macroinvertebrates on the SIBW. (a) A number of macroinvertebrates species (mean ± SD) were higher in S-F-E. (b) Principal component analysis (PCA) of the density and biomass of total macroinvertebrates in different vegetation were in the order of S-F-E > E > F-E. S-F-E, Submergent-flouting-emergent vegetation; F-E, Flouting-emergent vegetation; and E, Emergent vegetation.

In the SIBW, Emergent vegetation was mainly composed of* Typha orientalis*,* Phragmites australis*,* Polygonum persicaria,* and* Carex kirganica*; flouting vegetation was mainly composed of* Trapa manshurica*,* Nymphoides peltatum,* and* Lemna minor;* and submergent vegetation was mainly* Myriophyllum spicatum*,* Cladophora*,* Potamogeton distinctus,* and* Spirogyra*. In these three types of vegetation composition, the species composition and distribution of macroinvertebrates differed clearly (Figure 6). Generally, species of aquatic insects and mollusks dominated aquatic plant communities. The macroinvertebrates taxa were 36 in the types of Submergent-flouting-emergent vegetation (S-F-E), followed by the types of Emergent vegetation (E) and the types of Flouting-emergent vegetation (F-E), which were 20 and 19, respectively (Figure 6(a)). It can be noted from (Figure 6(b)) that PCA showed that the density and biomass values of macroinvertebrates also changed with the same tendency S-F-E, E and F-E. Only density differed significantly from one habitat type to another (p<0.05) (Table 7).

**Figure 7:**
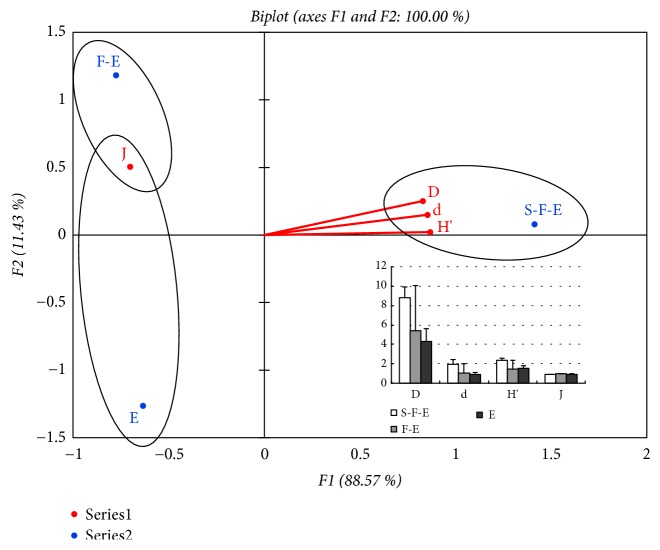
Principal component analysis (PCA) of biological indices comparison between three types of vegetation. The values of the index (D) and index (d) were the highest in the types of S-F-E vegetation, and next were in the types of F-E vegetation, and the two indices were lowest in the types of Emergent vegetation (E). While the values of the index (H′) were highest in the types of S-F-E vegetation, next were in Emergent vegetation (E), and the values of Pielou evenness index (J) were highest in the types of F-E vegetation and the lowest in the types of S-F-E vegetation. S-F-E, Submergent-flouting-emergent vegetation; F-E, Flouting-emergent vegetation; E, Emergent vegetation. D, Simpson diversity index; d, Margalef richness index; H′, Shannon-Weiner diversity index; J, Pielou evenness index. S-H, Silt-humus; M-G, Mud-gravel; M-S, Mud-sand.

From the study, the values of the Simpson diversity index (D) and Margalef richness index (d) were the highest in the types of S-F-E vegetation, and next were in the types of F-E vegetation, and the two indices were lowest in the types of Emergent vegetation (E). Whereas the values of the Shannon-Weiner index (H′) was highest in the types of S-F-E vegetation, next are in Emergent vegetation (E), and the values of Pielou evenness index (J) are highest in the types of F-E vegetation and the lowest showed in the types of S-F-E vegetation (Figure 7).

**Figure 8:**
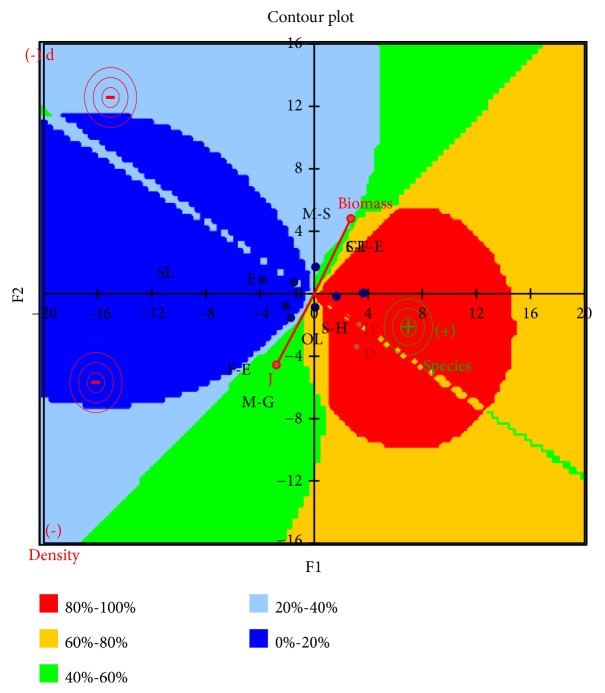
Principal component analysis (PCA) of preference mapping of macroinvertebrates distribution in the Sun Island Bund Wetland. Biotic index, density, and biomass comparison between three types of sediment, water state, and vegetation. The higher values of macroinvertebrates density, biomass, diversity, and number of species were recorded in the habitat types of silt-humus sediment, closed lentic area, and submerged-flouting-emergent vegetation. S-H, Silt-humus; M-G, Mud-gravel; M-S, Mud-sand. OL, Open lotic; SL, Seasonal lotic; CL, Closed lentic. S-F-E, Submergent-flouting-emergent vegetation; F-E, Flouting-emergent vegetation; E, Emergent vegetation. D, Simpson diversity index; d, Margalef richness index; H′, Shannon-Weiner diversity index; J, Pielou evenness index. S-H, Silt-humus; M-G, Mud-gravel; M-S, Mud-sand.

Finally, Figure 8 shows summary results of macroinvertebrate total biomass and density, number of species, and biotic index, when different habitat types were compared. The PCA results showed that the higher values of macroinvertebrates density, biomass, diversity, and number of species were recorded in the habitat types of silt-humus sediment, closed lentic area, and all aquatic vegetation.

## 4. Discussion

### 4.1. Relationship between Sediment and Macroinvertebrates

Macroinvertebrates spend most of their life on the bottom of aquatic ecosystems and therefore the sediment environment is important for determining the survival of different species of macroinvertebrates. The aquatic sediment provides a direct habitat and refuge to macroinvertebrates against enemies. Aquatic sediments material, particle size, and other factors directly affect the assemblage of benthic macroinvertebrates that reside in or on the sediments [26]. According to Yang and Chen [27] rocky substrate is suitable for macroinvertebrates species that can attach themselves. The silt or sand sediment is suitable for those species with burrowing habit such as some mollusks and crabs [28].

The number of species and the number of individuals per species are used as a basis for measuring the diversity of biological communities. The results of this study showed that the silty bottom had the highest number of macroinvertebrates species and diversity. This was probably attributed to the rich organic matter that provides a variety of food in a suitable environment [29]. The species of mollusks in the mud-gravel sediment were far less than in the silt or sand indicating that the mollusks are more likely to live in the sediment which has smaller particle size.

### 4.2. Relationship between the Water State and Macroinvertebrates

The flow of water and its connectivity is an important factor affecting the distribution of aquatic organisms [30]. From this study, macroinvertebrates attributes assessed showed that the closed lentic areas of the SIBW were the most suitable for the survival of macroinvertebrates. Because the closed lentic area is independently dominated by the marsh and a marsh is not influenced by external factors, the hydrological conditions are stable, making it easier for the macroinvertebrates to survive. By comparison, the seasonal lotic area, material circulation, and energy flow in this open area are faster and the water purification and the water quality are relatively better, so the number of the macroinvertebrates is relatively larger. Seasonal lotic areas registered less species of macroinvertebrates.

### 4.3. Effects of Vascular Aquatic Vegetation on Macroinvertebrates

Aquatic vegetation is the most important biological component of wetland ecosystems. It affects these ecosystems in a variety of ways. First, this vegetation plays a major role in the assimilation of nitrogen and phosphorus which reduces nutrient concentrations, improving self-purification of wetlands [31]. Second, aquatic vegetation provides habitats for aquatic plants [13]. A shift in the species composition of macrophyte type can likely have effects on the diversity, species richness, abundance, and biomass of macroinvertebrates. A well-established wetland with diverse vegetation will support a greater diversity, richness, and abundance of aquatic invertebrates [32]. This greater abundance of macroinvertebrates occurs because aquatic vegetation increases niche space and provides structural support and also higher food quality and protection from predators [14]. From the results, the structure of macroinvertebrate assemblages in SIBW appears to be strongly affected by the aquatic vegetation. The site of aquatic vegetation recorded the highest values of all macroinvertebrate attributes assessed in this study. This could be attributed to habitat diversity created by these species in different community types of vegetation. Different kinds of vegetation often support different kinds of macroinvertebrates [33, 34].

## 5. Conclusions

In this study, the effects of sediment type, water flow, and aquatic vegetation types on the assemblages of macroinvertebrates in the SIBW were assessed and their influence was discussed. However, other factors such as temperature, salinity, light, wave, tide, and human disturbance can also play an important role in the distribution of macroinvertebrates. Therefore, further studies on the influence of human activities and physic-chemical parameters on the structure of macroinvertebrate assemblages are needed.

## Figures and Tables

**Table 1:** Classification of habitat types.

Classification basis	Habitat Types		Stations
Sediment	Silt-humus	S-H	S1, S2, S4
	Mud-gravel	M-G	S3, S5, S6
	Mud-sand	M-S	S7, S8, S9

Water state	Open lotic	OL	S5, S6, S9
	Seasonal lotic	SL	S1, S3, S8
	Closed lentic	CL	S2, S4, S7

Composition of aquatic vascular vegetation	Submergent-flouting-emergent vegetation	S-F-E	S2, S4, S7
	Flouting-emergent vegetation	F-E	S1, S3, S5
	Emergent vegetation	E	S6, S8, S9

**Table 2:** Averages of physical and chemical parameters of the SIBW.

Parameters	WT(°C)	pH	NH_4_^+^(mg/L)	NO_2_^−^(mg/L)	PO_4_^3−^(mg/L)	N(mg/L)	Eh(mv)
Mean	28.000	7.721	0.511	0.017	0.714	1.611	-74.833
SD	1.095	0.282	0.176	0.014	0.152	0.831	16.764
p value among sediment types	0.967	0.603	0.630	0.169	0.803	0.379	0.572
p value among water state types	0.177	0.801	0.026*∗*	0.078	0.249	0.001*∗*	0.819
p value among vegetation types	0.176	0.827	0.094	0.195	0.114	<0.001*∗*	0.826

*∗*: represents a significant difference (*α* = 0.05).

**Table 3:** Species of macroinvertebrates and its distribution in SIBW.

Orders	Families	Species	Sediment			Water state			Composition of aquatic vascular vegetation		
			S-H	M-G	M-S	OL	SL	CL	S-F-E	F-E	E
	**Aquatic insect**										
Diptera	Chironomidae	*Cricotopus albiforceps*	+	+	+	+		+	+	+	+
		*C. triannulatus*		+	+	+		+	+		+
		*C. annulator*	+					+	+		
		*Eukiefferiella claripennis*	+	+		+		+	+	+	
		*E. fuldensis*	+					+	+		
		*E. fittkaui*	+					+	+		
		*E. lobifera*	+	+		+		+	+	+	
		*E. gracei*			+	+					+
		*Micropsectra chuzeprima*	+					+	+		
		*Orthocladius frigidus*			+	+					+
		*O. thienemanni*		+		+				+	+
		*Polypedilum flavum*	+					+	+		
		*P. nubifer*	+	+	+	+		+	+		+
		*P. albicorne*	+		+	+		+	+		+
		*P. nubeculosum*	+					+	+		
		*P. asakawaense*		+	+	+		+	+	+	
		*P. pedestre*			+			+	+		
		*Rheocricotopus effusus*	+	+	+	+		+	+	+	+
Hemiptera	Belostomatidae	*Kirkaldyia deyrollei*	+					+	+		
	Nepidae	*Ranatra chinensis*	+					+	+		
Odonata	Gomphidae	*Sieboldius* sp.	+		+	+		+	+		+
		*Stylurus flavipes*	+					+	+		
Trichoptera	Sericostomatidae	*Gumaga okinawaensis*	+					+	+		
	Polycentropodidae	*Polycentropus* sp.		+		+				+	
	Hydropsychidae	*Hydropsyche ulmeri*	+				+			+	
	Rhyacophilidae	*Rhyacophila* sp.		+		+					+
Ephemeroptera	Ephemeridae	*Ephemera* sp.			+		+				+
	Heptageniidae	*Heptagenia* sp.		+		+				+	
	Ephemerellidae	*Serratella* sp.		+		+				+	
	Leptophlebiidae	*Paraleptophlebia* sp.	+					+	+		
		**Gastropoda**									
Basommatophora	Lymnaeidae	*Radix auricularia*	*+*					+	+		
		*R. swinhoei*	*+*	+	+	+		+	+	+	+
		*R. ovate*	*+*					+	+		
		*R. plicatula*						+	+		
		*Radix lagotis*			+	+					+
		*Galba pervia*	+		+	+		+	+		+
		*G. truncatula*	+					+	+		
	Planorbidae	*Gyraulus convexiusculus*		+			+			+	
		*G. albus*	+					+	+		
	Viviparidae	*Cipangopaludina ussuriensis*			+			+	+		
		*C. cahayensis*	+		+	+		+	+		+
		*Bellamya aeruginosa*	+		+	+		+	+		+
		*B. purificata*	+		+	+	+	+	+	+	+
		*Viviparus chui*			+			+	+		
	Bithyniidae	*Bithynia fuchsiana*	+	+	+	+	+			+	+
		*Parafossarulus striatulus*			+	+	+				+
	**Annelida**										
Tubificidae	Tubificidae	* Limnodrilus claparedeianus*			+			+	+		
Rhynchobdellida	Glossiphoniidae	*Helobdella nuda*			+			+	+		
		*Glossiphonia heteroclita*		+			+			+	
		*Whitmania pigra*		+		+				+	
	**Crustacea**										
Decapoda	Palaemonidae	* Exopalaemon annandalei*	+	+	+	+	+	+	+	+	+
		*Palaemon myadii*		+		+				+	
Amphipoda	Gammaridae	*Gammarus pulex*		+		+				+	

+: represents this species occurs in the area.

**Table 4:** Averages of various biological indexes of macroinvertebrates in SIBW.

	Density (ind./m^2^)	Biomass (g/m^2^)	D	d	H′	J
Mean	340.889	390.248	6.156	1.284	1.783	0.934
	275.295	484.167	3.217	0.764	0.645	0.033
Range	26–871	21.37–1543.65	2–10.756	0.307–2.511	0.693–2.558	0.885–1

**Table 5:** Biotic index comparison between three types of sediment (mean±SE).

	Number of species	Density (ind. /m^2^)	Biomass (g/m^2^)	D	d	H′	J
S-H	31.00±7.02	472.33±364.54	358.09±114.45	7.26±3.43	1.65±0.97	2.04±0.67	0.91±0.02
M-G	20.00±5.50	164.66±126.26	31.07±15.19	6.17±4.39	1.19±0.91	1.66±0.90	0.96±0.02
M-S	24.00±6.02	385.66±280.73	781.57±706.80	5.02±2.56	1.00±0.47	1.63±0.49	0.92±0.01

p value	0.727	0.423	0.164	0.751	0.637	0.743	0.031*∗*

**Table 6:** Biotic index comparison between three types of water state.

	Number of species	Density (ind./m^2^)	Biomass (g/m^2^)	D	d	H′	J
OL	**28.00**	**294.66**±112.08	**241.16**±357.38	**6.79**±3.56	**1.36**±0.69	**1.91**±0.52	**0.94**±0.02
SL	**8.00**	**91.00**±65.00	**163.74**±149.25	**2.89**±0.78	**0.52**±0.19	**1.08**±0.34	**0.95**±0.04
CL	**36.00**	**637.00**±240.76	**765.83**±682.48	**8.77**±1.11	**1.96**±0.49	**2.35**±0.18	**0.90**±0.02

p-value	0.023*∗*	0.015*∗*	0.283	0.043*∗*	0.034*∗*	0.017*∗*	0.297

*∗*: represents a significant difference (*α* = 0.05).

The value is mean ± *SD*, except the number of species.

**Table 7:** Biotic index comparison between three types of vegetation.

	Number of species	Density (ind. /m^2^)	Biomass (g/m^2^)	D	d	H′	J
S-F-E	**36.00±**3.79	**637.00**±240.76	**765.84**±682.48	**8.78**±1.11	**1.97**±0.50	**2.35±0.18**	**0.91**±0.02
F-E	**19.00**±5.86	**151.67**±123.56	**130.76**±164.75	**5.40**±4.70	**1.01**±0.99	**1.48**±0.90	**0.96**±0.04
E	**20.00**±6.25	**234.00**±165.97	**274.15**±334.56	**4.29**±1.31	**0.87**±0.24	**1.51**±0.29	**0.93**±0.02

p-value	0.314	0.037*∗*	0.268	0.22	0.162	0.177	0.099

*∗*: represents a significant difference (*α* = 0.05).

The value is mean±*SD*, except the number of species.

## Data Availability

The data used to support the findings of this study are included within the article.
